# Egg Yolk Granule Nanoparticles Promote Longitudinal Bone Growth in HFD-Obese Mice

**DOI:** 10.3390/foods14173109

**Published:** 2025-09-05

**Authors:** Wanyu Xue, Chunhui Yu, Haodong Liu, Jingxuan Zhang, Bo Li

**Affiliations:** College of Food Science and Nutritional Engineering, China Agricultural University, Beijing 100083, China; 18501230199@163.com (W.X.); 13704972137@163.com (C.Y.); liu18742060205@163.com (H.L.); jingxuanzhang103@gmail.com (J.Z.)

**Keywords:** egg yolk, digestibility, obesity model, bone metabolism

## Abstract

The relationship between obesity and bone development remains uncertain and requires further study. Egg yolk granules (EYGs), due to their high content of phosvitin (PV), are speculated to have the potential to promote bone growth. And EYGs nanoparticles (EYG NPs) may help improve their digestibility. This study aimed to evaluate the effects of EYG NPs on longitudinal bone growth in high-fat diet-induced obese mice. EYG NPs were prepared by treating EYGs with (NaPO_3_)_6_ and ultrasonication, then characterized. The simulated gastrointestinal digestion experiment indicated that the modification of EYG significantly enhanced the digestibility of PV. After 12 weeks of intervention, body growth indicators, serum bone metabolism markers, tibial length, bone mineral density (BMD) and growth plate height were measured. In obesity model, the body length increased, while serum ALP activity decreased, and BMD showed no differences compared to those in Normal group. High-dose EYG NPs supplementation promoted longitudinal bone growth of obese mice, as evidenced by increased tibial length, elevated serum ALP activity, and enhanced growth plate height, while maintaining BMD. These findings suggest that EYG NPs have the potential of promoting longitudinal bone growth among the adolescent obese population.

## 1. Introduction

Numerous studies have demonstrated that unbalanced dietary patterns are closely associated with the development of obesity. In particular, high-fat diets can lead to excessive energy intake and promote adipose tissue accumulation. Long-term consumption of high-fat diets may also increase the risk of type 2 diabetes and cardiovascular diseases [1]. In recent years, the incidence of obesity in adolescent populations has shown a consistent upward trend over recent years [2]. As this age group is in a critical period of growth and development, obesity may interfere with normal growth patterns [3]. It can impair skeletal development, potentially leading to incomplete bone maturation, growth retardation, and an increased risk of fractures and osteoarthritis. These effects may have long-term consequences on overall health throughout the lifespan [4,5]. Longitudinal bone growth, which ultimately determines adult height, is regulated by the activity of the growth plate located near the epiphysis of long bones. Endochondral ossification involves chondrocyte proliferation, hypertrophy, and calcification within the growth plate, and is regulated by multiple factors such as IGF, TGF-β, thyroid hormones, Sox9, and Runx [6]. The proliferation and hypertrophy of chondrocytes within this cartilage region drive the elongation of bones [7], and thus are closely related to height increase during adolescence.

Recently, food-derived proteins and bioactive peptides have attracted growing attention for their potential to improve bone health. Egg yolk is rich in phosvitin (PV), one of the most highly phosphorylated proteins. PV has the effect of improving bone health in experimental animals by promoting osteogenic differentiation and enhancing bone formation. Fernando et al. [8] prepared water-soluble peptides derived from egg yolk which have been shown to promote preosteoblast proliferation and their differentiation into osteoblasts, thereby enhancing mineralization activity. Chakrabarti et al. [9] reported that phosphopeptides (PPPs) derived from hydrolyzed PV exhibited strong effects on promoting osteoblast proliferation, differentiation and mineralization, thereby contributing to improve bone health. Zhao et al. [10] prepared functional PPPs from PV and found that PPPs showed higher calcium-chelating capacity and greater enhancement of osteoblast differentiation and mineralization compared to PV.

Egg yolk granules (EYGs) are byproducts during the extraction of egg yolk immunoglobulins. EYGs consist of approximately 70% HDLs, 16% PV, and 12% low-density lipoproteins (LDLs) [11]. Due to their high content of HDLs and PV, EYGs are hypothesized to promote bone health and represent a promising source for developing functional foods. However, EYGs are formed by the tight association of PV and HDLs through calcium phosphate bridges, and some LDL vesicles are embedded within it, resulting in formation of spherical complexes with a diameter of approximately 0.3–2 μm [11,12]. The dense structure is the primary reason for the poor solubility of EYGs which restricts their utilization. Modified EYGs have been applied in the food industry such as emulsifiers [13,14] and nutrient delivery nanocarriers [15,16,17,18]. Moreover, studies indicated that adjusting pH and ionic strength could partially improve the solubility and digestibility of EYGs [19]. Therefore, we speculate that highly soluble EYGs nanoparticles (EYG NPs) can expose more protein cleavage sites for digestive enzymes, thereby enhancing the bioavailability of PV and HDLs.

Extensive animal research has shown that high-fat diets effectively induce obesity models, which has led to their widespread use in studying metabolic disorders related to obesity [20]. However, most studies on longitudinal bone growth rely on adolescent rodent models with relatively short durations (3–4 weeks), lacking long-term intervention and dynamic observation. Therefore, this study aims to evaluate the effects of EYG NPs on longitudinal bone growth using a high-fat diet-induced obesity mouse model starting at 6 weeks of age for 12 weeks. This research not only uncovered the new functions of EYG NPs but also provided new solutions to improve bone health for obese individuals.

## 2. Materials and Methods

### 2.1. Materials and Reagents

Fresh eggs were obtained from Qiuxian Agricultural Technology Co., Ltd. (Handan, China). The BCA protein assay kit was purchased from Solarbio Science & Technology Co., Ltd. (Beijing, China). The calcium detection kit was obtained from Beyotime Biotechnology (Shanghai, China). (NaPO_3_)_6_, NaOH, HCl and sterile normal saline were all provided by Sinopharm Chemical Reagent Co., Ltd. (Shanghai, China). 4% paraformaldehyde solution was obtained from Beijing Baide Biotechnology Co., Ltd. (Beijing, China). All other chemicals used in this study were of analytical grade or better.

### 2.2. Preparation of EYGs

Fresh eggs were separated to obtain yolks, and residual egg white was gently removed using filter paper. The yolk membrane was punctured, and the contents were collected and homogenized. The yolk was diluted 1:10 (*v*/*v*) with deionized water, and the pH was adjusted to 5.0–5.2 using 1.0 mol/L HCl. After overnight storage at 4 °C, the mixture was centrifuged at 10,000× *g* for 15 min at 4 °C. The precipitate was washed repeatedly with deionized water to remove surface impurities, then diluted with 0.17 mol/L NaCl solution (1:3, *w*/*v*), shaken at 40 °C for 1 h, and centrifuged at 10,000× *g* for 15 min at 4 °C. The resulting precipitate was collected as EYGs.

### 2.3. Nutrient Composition of EYGs

Moisture was measured by gravimetric analysis after oven-drying at 105 °C by the Kjeldahl method. Fat content was extracted with petroleum ether and measured gravimetrically. Ash content was calculated after incineration. Cholesterol was quantified by GC after saponification, solvent extraction, concentration, and redissolution.

### 2.4. Amino Acid Component Analysis

Amino acid contents were analyzed based on Hou et al. [21] with minor modifications. The sample (6 mg) was hydrolyzed at 110 °C for 24 h using 6 mol/L HCl. The hydrolysate was evaporated, redissolved in 0.1 mol/L HCl, derivatized with phenyl isothiocyanate, extracted with hexane, and analyzed by capillary HPLC (Thermo Fisher Scientific, Waltham, MA, USA).

### 2.5. Preparation of EYG NPs by Treatment EYGs with (NaPO_3_)_6_ and Ultrasonic

A 2.0% (*w*/*v*) dispersion of EYGs was prepared, and the pH was adjusted to 7.0, 7.5, or 8.0, respectively. Then 100 mg/mL of (NaPO_3_)_6_ solution was then added to achieve final concentrations of 0.1%, 0.2%, 0.3%, 0.4%, and 0.5% (*w*/*w*) relative to the mass of the granules, followed by magnetic stirring for 2 h. Subsequently, samples were subjected to ultrasonic treatment at 200 W for 5, 10, 15, 20, 25, or 30 min. All samples were diluted to 1.0 mg/mL prior to particle size measurement. The untreated sample was named as C, pH-adjusted sample was named as C′, phosphate-treated sample was named as P, ultrasound-treated sample was named as U, the sample with both treatments was named as PU.

### 2.6. Measurement of Particle Size and Zeta Potential

EYGs and EYG NPs were appropriately diluted and then analyzed for particle size using a NanoBrook 90 Plus PALS analyzer (Brookhaven Instruments, Nashua, NH, USA).

### 2.7. Soluble Protein Concentration

Samples were diluted to a final concentration of 2 mg/mL and centrifuged at 10,000× *g* for 15 min at 4 °C. The resulting supernatants were used to determine the soluble protein concentration, and which was quantitatively analyzed using a BCA protein assay kit (PC0020, Solarbio, Beijing, China).

### 2.8. Calcium Ion Content

Samples (5.0 mg/mL) were centrifuged at 10,000× *g* for 15 min at 4 °C, and the supernatants were collected. Calcium content was measured with a calcium ion color detection kit (S1063S, Beyotime, Shanghai, China).

### 2.9. Turbidity Measurement

Samples were diluted to 1.0 mg/mL and the absorbance at 600 nm was measured using a UV-Vis spectrophotometer. Deionized water was used as the blank.

### 2.10. Surface Hydrophobicity

Samples were diluted to 2.0 mg/mL. Then, 0.2 mL of 1.0 mg/mL bromophenol blue (BPB) was added to 1 mL of sample, vortexed for 10 min, and centrifuged at 10,000× *g* for 15 min. The supernatant (20-fold diluted) was measured at 595 nm [22]. The amount of bound BPB was calculated as:

BPB bound (μg) = 200 μg × (ODcontrol − ODsample)/ODcontrol(1)

### 2.11. Transmission Electron Microscopy (TEM)

Sample microstructure was observed using TEM (JEM 1200EX, JEOL Corporation, Tokyo, Japan). Diluted samples were dropped on copper grids and imaged under varying magnifications.

### 2.12. Determination of Phosphorus Content and Digestibility

For the pre-treatment of EYG NPs, the solution of EYG NPs was placed in dialysis bags (MWCO 1 kDa) and dialyzed against deionized water at 4 °C under gentle stirring. The water was replaced every 2–3 h for the first 12 h and every 6–12 h thereafter, for a total of 24–36 h. Subsequently, the sample was freeze-dried.

In vitro gastrointestinal digestion was performed according to the method described by Song et al. [23], with slight modifications. Briefly, 2.5 g of sample was dissolved in 200 mL of 0.01 mol/L HCl (pH 2.0), followed by the addition of 2% pepsin (E/S, *w*/*w*). The mixture was shaken at 37 °C, 130× *g* for 2 h to simulate gastric digestion. The pH was then adjusted to 7.0, and trypsin was added at 2% (E/S). The sample was further incubated under the same conditions for 2 h to simulate intestinal digestion. Enzymes were inactivated by boiling for 10 min. The digested sample was centrifuged (5000× *g*, 15 min), and the collected supernatant was freeze-dried and preserved at −80 °C until analysis.

The phosphorus content was quantified using the molybdenum blue colorimetric method, following the procedure described by Li et al. [24] with slight modifications, with KH_2_PO_4_ as the standard solution.

Digestibility was calculated as:

(2) $$Digestibility\left(\%\right)=\frac{m0-m1}{m0}\times 100\%$$

where m_0_ is the phosphorus content before digestion and m_1_ after digestion.

### 2.13. Animal Experiments and Treatments

Animal experiments were approved by the Welfare Committee of the Center of Experimental Animal, Beijing, China (AW90904202-4-2). All procedures were conducted in strict accordance with the ARRIVE guidelines. A total of forty male C57BL/6J mice (certification No. SYXK (Beijing) 2021-0012) aged 6 weeks and weighing 20 ± 2 g were obtained from Beijing Vital River Laboratory Animal Technology Co., Ltd. (Beijing, China). Mice were housed under SPF conditions. After acclimation, 40 mice were randomly divided into 4 groups (Table S1) using a random number generator and fed for 12 weeks. Detailed group assignments and dietary interventions were as follows. The normal group was fed with low-fat diet (10% total fat content), the model group was fed with a high-fat diet. The high-fat diet consisted of 20% energy percent (En%) carbohydrates, 20 En% protein, and 60 En% fats [25,26] (Tables S2–S4). To investigate the effect of EYG NPs on bone growth in obese children, EYG NPs were added to a high-fat diet at low (EL, 0.95% *w*/*w*) and high (EH, 1.90% *w*/*w*) doses in the animal study, resulting in four treatment groups: normal control group (N group), high-fat diet model group (M group), high-fat diet with low-dose EYG NPs group (EL group), high-fat diet with high-dose EYG NPs group (EH group). Mice were fasted for 12 h at the end of the 12-week experiment, then they were sacrificed following deep anesthesia with 2.5% Avertin until cessation of heartbeat and respiration. During histological processing and quantitative analysis, investigators were blinded to the group allocation.

### 2.14. Body Weight, Total White Adipose Weight and Organ Index

Body weight was recorded weekly. After mice were sacrificed, liver, adipose tissues (including epididymal fat, subcutaneous fat, and perirenal fat), and other major organs were carefully dissected and weighed. The organ index was calculated using the following formula:

Organ index = Organ weight (mg)/Body weight (g) × 100%(3)

### 2.15. Body Length and Tibial Length

After anesthesia, the mice were carefully placed next to a ruler for photography. The body length of each mouse was measured as the linear distance from the nose to the tail base. The right tibia was carefully excised after the mice were sacrificed, and the surface muscle tissue was removed. The tibial lengths were measured using a caliper (accuracy: 0.01 mm) from the proximal to the distal end. Each measurement was performed in triplicate, and the average value was recorded. Observers were blinded to the experimental groups during measurement to ensure unbiased results.

### 2.16. Bone Mineral Density

Left femurs were collected, wrapped in saline-soaked gauze, and stored at 4 °C. Bone mineral density was measured using DEXA (Hologic Discovery A, Marlborough, MA, USA). Daily calibration of the instrument was conducted using a standard phantom. The bone mineral density of the whole bone was measured.

### 2.17. Tissue Collection and H&E Staining of the Bone Growth Plate

The right tibias were fixed in 4% paraformaldehyde, decalcified with EDTA, embedded in paraffin, and subsequently stained with hematoxylin and eosin (H&E) for histological analysis. Growth plate height was analyzed using SlideViewer 2.5 software. For each tibia, three evenly spaced regions along the growth plate were selected, and measurements were performed in triplicate. The average growth plate height was calculated for each mouse.

### 2.18. Serum Biochemical Parameters

After anesthesia, blood samples were then collected and stored in 1.5 mL centrifuge tubes. After standing at room temperature for approximately 2 h, the samples were centrifuged (4 °C, 3500× *g*, 10 min) to obtain serum. The serum was analyzed for calcium, phosphorus, and ALP activity using a clinical biochemistry analyzer (Hitachi, Tokyo, Japan).

### 2.19. Statistical Analysis

All data were expressed as either mean ± SD or mean ± SEM, depending on the type of analysis. For comparisons involving more than two groups, a one-way ANOVA followed by Tukey’s post hoc test was performed. Significant differences between groups are indicated in figures and tables using different lowercase superscript letters. The results were expressed as mean ± SD. For animal experiments comparing only two groups, *t*-test was applied. In these cases, data are presented as mean ± SEM. Statistical analyses were conducted using software SPSS 22.0, and a value of *p* < 0.05 was considered statistically significant. Figure constructions were performed using GraphPad Prism ver. 6.01 (GraphPad Software, San Diego, CA, USA).

## 3. Results and Discussion

### 3.1. Nutrient Content and Amino Acid Composition of EYGs

The protein content in EYGs reached 53.6%, with low fat and cholesterol levels, indicating potential as a nutritious food ingredient (Table 1). The amino acid composition of the EYGs was analyzed to evaluate their protein quality (Table 2). Among all amino acids, leucine showed the highest content (16.82 g/100 g), followed by lysine (9.50 g/100 g). EYGs have a well-balanced amino acid profile and could serve as a high-quality protein source in functional foods.

### 3.2. The Particle Size of EYGs Under Different Treatments

**Figure 1:**
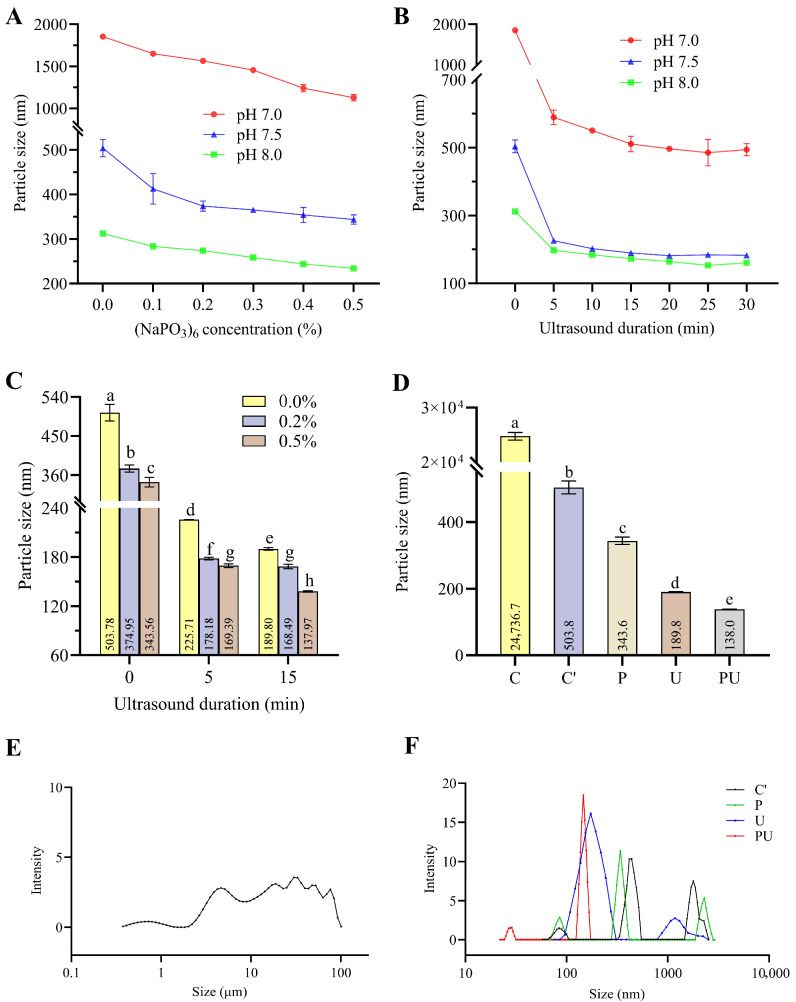
Particle size and distribution of egg yolk granules (EYGs) under different treatment conditions. (**A**) Effect of (NaPO_3_)_6_ concentration and pH value on the particle size of EYGs. (**B**) Effect of ultrasound duration and pH value on the particle size of EYGs. (**C**) Combined effect of (NaPO_3_)_6_ concentrations and ultrasound durations. (**D**) Average particle size under different treatment conditions. (**E**) Particle size distribution of untreated egg yolk granules. (**F**) Particle size distribution under different treatment conditions. Values are expressed as mean ± SD (*n* = 3). Statistical analysis was performed using one-way ANOVA followed by Tukey’s post hoc test. Different letters indicate significant differences (*p* < 0.05). Five treatment groups were as follows: C, Control; C′, pH7.5 group; P, phosphate treatment group; U, ultrasound treatment group; and PU, phosphate combined with ultrasound treatment group.

The effects of (NaPO_3_)_6_, pH, ultrasound, and their combination on particle size of EYGs were investigated. The results indicated that the particle size of EYGs gradually reduced when (NaPO_3_)_6_ concentration and pH value increased (Figure 1A), which can be attributed to the Ca^2+^-chelating ability of (NaPO_3_)_6_ that disrupts the structural integrity of EYGs. The particle size of EYGs decreased significantly after 15 min of ultrasound duration, with no further significant change observed when extended to 20 min (Figure 1B), indicating that short-term ultrasound (15 min) effectively disrupts large particles. Geng et al. [27] demonstrated that high-intensity ultrasound significantly reduced the average particle size of egg yolk granules from 289.4 nm (untreated) to 181.4 nm (270 W treatment). Furthermore, elevated pH effectively decreased the particle size of EYGs (Figure 1A,B), which is consistent with Anton’s findings [28].

The combined effects of (NaPO_3_)_6_ concentration and ultrasound time on the particle size of EYGs at pH 7.5 were further investigated. Combined treatment of phosphate and ultrasound exhibited a synergistic effect in reducing the particle size of EYGs (Figure 1C). The particle size of EYGs was minimized (138 nm) under the combined treatment of 0.5% (NaPO_3_)_6_ and ultrasonic treatment for 15 min at pH 7.5 (PU), significantly smaller than that of the untreated group (C) and single-treatment groups (C′, P, and U) (Figure 1D). Consistently, over 90% of the particles in the PU group were distributed within the 100–200 nm size range (Figure 1F), which was significantly smaller compared to the natural particle size of EYGs (Figure 1E). These results suggested that EYG NPs were fabricated through a combined treatment of native EYGs with (NaPO_3_)_6_ and ultrasonication.

### 3.3. Turbidity, Zeta-Potential and Surface Hydrophobicity of EYGs and EYG NPs

**Figure 2:**
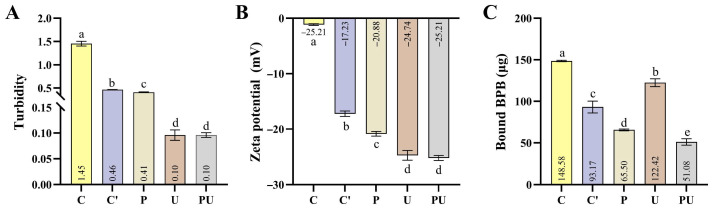
The effect of (NaPO_3_)_6_ combined with ultrasound on the turbidity, zeta-potential and surface hydrophobicity of egg yolk granules. (**A**) Turbidity; (**B**) Zeta-potential; (**C**) Surface hydrophobicity. The figure includes five treatment groups: Control, C′ (pH7.5 group), P (phosphate treatment group), U (ultrasound treatment group), and PU (phosphate combined with ultrasound treatment group). Values are expressed as mean ± SD (*n* = 3). Statistical analysis was performed using one-way ANOVA followed by Tukey’s post hoc test. Data with different letters indicate statistically significant differences (*p* < 0.05).

Turbidity reflects particle aggregation in suspensions, and lower values suggest better dispersion [29]. The treatment of phosphate and ultrasound significantly reduced the turbidity of EYGs suspensions (0.096) compared to the C group (1.45) (Figure 2A). In addition, under phosphate treatment, sodium ions can replace Ca^2+^, disrupting the calcium-phosphate bridges that maintain the structural integrity of EYGs. This disruption leads to the disassembly of the granules, resulting in the dissolution of PV and the release of internal components, thereby decreasing the turbidity of the solution [30].

Zeta potential indicates particle surface charge, and higher absolute values mean stronger electrostatic repulsion. In this study, the absolute zeta potential increased under different treatment conditions, which was attributed to the disaggregation of EYGs and the consequent exposure of negatively charged phosphoserine residues (Figure 2B). Native EYGs showed a near-zero zeta potential, indicating minimal surface charge and a high degree of aggregation. Phosphate treatment increased the zeta potential (from −1.13 mV to −20.88 mV) due to the chelation of Ca^2+^ and subsequent exposure of negatively charged phosphate groups. Ultrasonic treatment had the most pronounced effect (from −1.13 mV to −23.95 mV), likely due to the greater disruption of particle structure, exposing more charged groups and enhancing electrostatic repulsion—consistent with the observed reduction in turbidity.

Surface hydrophobicity was assessed based on the binding capacity of BPB. EYGs in the P and C′ groups showed significantly lower BPB binding compared to the C group. In contrast, BPB binding was increased in the U group (compared to the C group), possibly due to ultrasound-induced protein unfolding and exposure of hydrophobic groups. Even so, the lowest BPB binding (51.1 μg) was observed in the PU group (Figure 2C). This might be attributed to the formation of self-assembly of the nanoparticles, which enhanced internal hydrophobic interactions and increased the overall hydrophilicity of the particles.

### 3.4. Soluble Protein Content and Ca^2+^ Release of EYGs and EYG NPs

The soluble protein content was significantly increased in all treatment groups, with the PU group exhibiting an approximately sixfold increase compared to the C group (Table 3). The results indicated that PU treatment caused depolymerization of egg yolk granules and the release of internal soluble proteins. Furthermore, (NaPO_3_)_6_ chelated Ca^2+^ from the calcium phosphate bridges, leading to an increased Ca^2+^ concentration in the solution.

### 3.5. Microstructure of EYGs and EYG NPs

**Figure 3:**
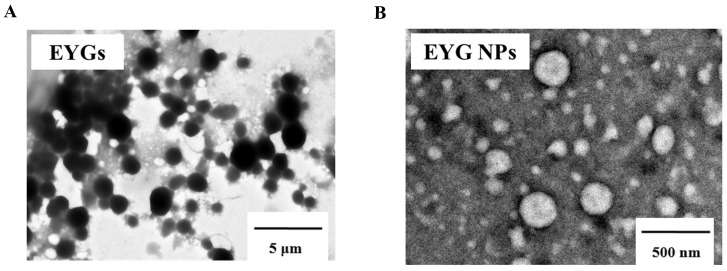
Microstructure of EYGs and EYG NPs. (**A**) EYGs (Unprocessed group treatment). (**B**) EYGs treated with (NaPO_3_)_6_ combined with ultrasound.

The microstructural features of EYGs samples were investigated using TEM. As shown in Figure 3, the untreated granules (A) exhibited irregular shapes with large particle sizes and obvious aggregation. After combined treatments, EYGs obtained showed a significant reduction in size to the nanoscale and appeared as uniformly dispersed spherical particles (B).

### 3.6. In Vitro Digestibility of EYGs and EYG NPs

EYGs are rich in PV, a highly phosphorylated protein. Measuring phosphorus content before and after simulated digestion provides an indirect assessment of the digestibility of proteins, particularly phosvitin-derived PPPs. In this study, the PV digestibility in EYG NPs reached 16.88%, which was significantly higher than that of native EYGs (12.04%, *p* < 0.05) (Table 3). These results indicated that the depolymerization of the rigid structure of EYGs improved the digestibility of PPPs, although this result based on simulated gastrointestinal digestion did not take into account the subsequent processes including intestinal transport, cellular uptake, first-pass metabolism, and systemic distribution [31,32].

### 3.7. EYG NPs Promoted Longitudinal Bone Growth in Obesity Model of Mice

#### 3.7.1. Body Weight, Body Length and Organ Indexes

**Figure 4:**
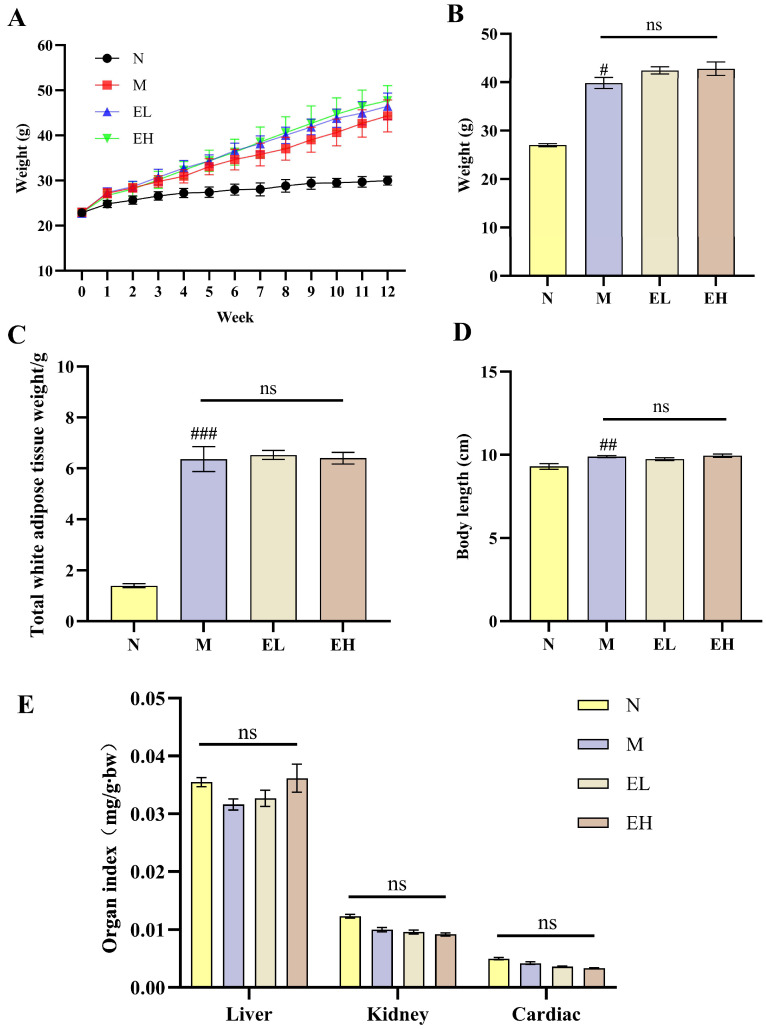
Effects of egg yolk nanoparticles on mice growth parameters. (**A**) Weight changes in mice during the 12-week experiment; (**B**) Body weight of mice at week 12; (**C**) Total white adipose weight; (**D**) Body length of mice after 12 weeks. (**E**) Organ index (liver, kidney and cardiac). The four treatment groups in the figure: N (normal control group), M (high-fat diet model group), EL (high-fat diet with 0.95% egg yolk nanoparticles group), EH (high-fat diet with 1.90% egg yolk nanoparticles group). Values are expressed as mean ± SEM (*n* = 10). Statistical significance was determined using a *t*-test: #, significant difference compared to N group at *p* < 0.05; ## significant difference compared to N group at *p* < 0.01; ### extremely significant difference compared to N group at *p* < 0.001; ns, no statistically significant difference at *p* > 0.05.

After one week of high-fat diet feeding, mice in the model group (M) appeared a significantly higher body weight (Figure 4A), and the body weight and total white adipose tissue mass at 12 weeks were much higher than those in the normal diet group (N) (Figure 4B,C), indicating successful establishment of the obesity model. During the 12-week intervention with EYG NPs, the body weight of two groups (EL, EH) showed the same upward trend as M group (Figure 4A). In terms of body length, mice in the M group were significantly taller than those in the N group, and similar findings were observed in the two treatment groups (*p* < 0.01; Figure 4D).

As shown in Figure 4E, intake of EYG NPs showed no significant differences in liver, kidney, or heart indexes compared to N or M group (*p* > 0.05). Organ index is an important indicator of systemic tolerance to test substances. These findings suggest that oral administration of EYG NPs at the tested dosages had no apparent safety risk to the organism.

#### 3.7.2. BMC, BMD, Longitudinal Bone and Growth Plate

**Figure 5:**
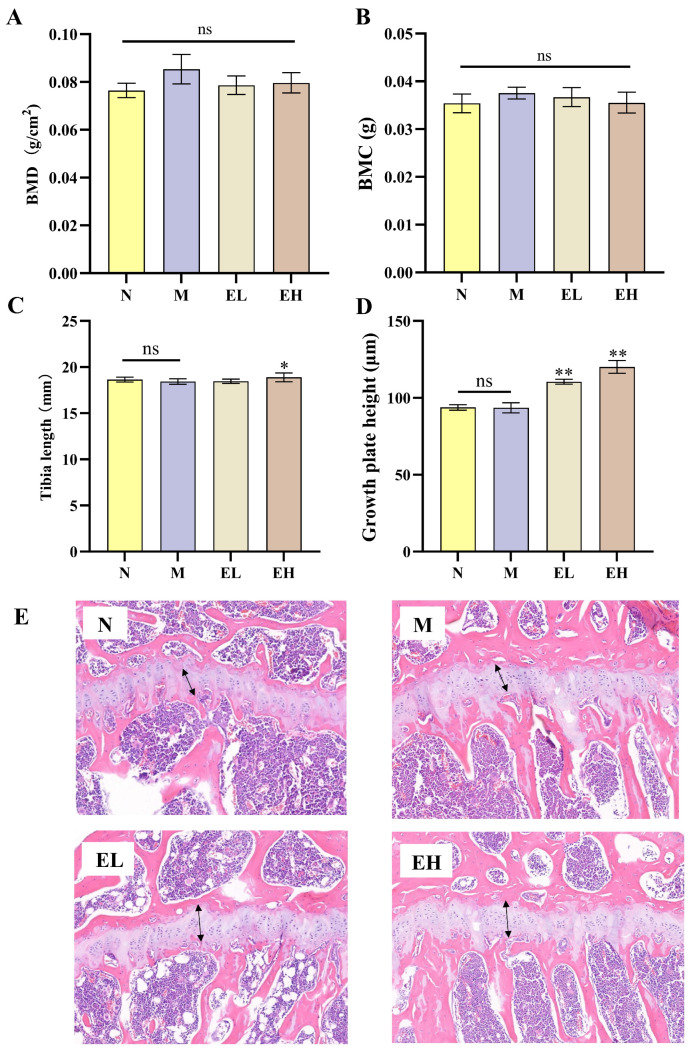
Effect of egg yolk nanoparticles on bone growth parameters in mice. (**A**) Femur bone mineral density (BMD); (**B**) Femur bone mineral content (BMC); (**C**) Tibia length after 12 weeks of intervention; (**D**) Growth plate height; (**E**) H&E staining of the growth plate (10×). The arrow indicates the height of the growth plate. The four treatment groups in the figure: N (normal control group), M (high-fat diet model group), EL (high-fat diet with 0.95% egg yolk nanoparticles group), EH (high-fat diet with 1.90% egg yolk nanoparticles group). Values are expressed as mean ± SEM (*n* = 3). Statistical significance was determined using a *t*-test: *, **, significant difference compared to M group at *p* < 0.05 or *p* < 0.01, respectively; ns, means no statistically significant difference (*p* > 0.05).

Bone mineral density (BMD), which reflects the amount of bone mineral content (BMC), serves as a core marker of bone health [33]. As shown in Figure 5A,B, although the M group exhibited the highest BMD and BMC values, no significant differences were observed among N, M, EL and EH groups (*p* > 0.05), indicating that EYG NPs intervention did not influence BMC or BMD.

Regarding longitudinal bone growth (Figure 5C), the EH group showed a significant increase in tibial length compared to the M group (*p* < 0.05), suggesting that high-dose EYG NPs can promote bone growth in mice.

Longitudinal bone growth ultimately determines adult height, and the growth plate is the site of longitudinal bone elongation. The growth plate is divided into resting, proliferative, maturation, and hypertrophic zones, which coordinate chondrocyte proliferation and enlargement, ultimately contributing to bone lengthening [34]. The morphological examination of the bone growth plate was illustrated in Figure 5E. The growth plate in tibias was stained a grayish white with H&E (black arrows). After quantitative analysis using SlideViewer 2.5 software, the growth plate height of each group was shown in Figure 5D. There was no significant difference in the height of the growth plates between the N and M group (*p* > 0.05); however, in comparison to the M group, the EL and EH groups exhibited a significantly increase in the height of the growth plate (*p* < 0.01). Specifically, the increase was 18.1% for the EL group and 28.4% for the EH group (Figure 5D). Longitudinal bone growth primarily depends on the development of the growth plate at the epiphyseal end of long bones [34], this study indicated that EYG NPs may promote longitudinal bone growth by increasing the height of the growth plate.

#### 3.7.3. Serum Bone Markers

**Figure 6:**
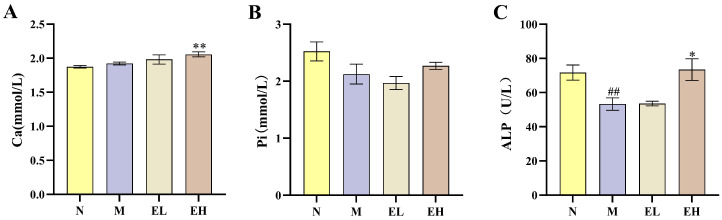
Effect of egg yolk nanoparticles on serum biomarkers of mice. (**A**) Serum calcium; (**B**) Serum phosphorus; (**C**) Serum alkaline phosphatase (ALP) activity. The four treatment groups in the figure: N (normal control group), M (high-fat diet model group), EL (high-fat diet with 0.95% egg yolk nanoparticles group), EH (high-fat diet with 1.90% egg yolk nanoparticles group). Values are expressed as mean ± SEM (*n* = 7). Statistical significance was determined using a *t*-test. *, a significant difference compared to M group (*p* < 0.05); **, a highly significant difference compared to M group (*p* < 0.01); ##, a highly significant difference compared to N group (*p* < 0.01).

Serum calcium and phosphorus are key mineral components of the skeletal system and closely related to bone metabolism. As shown in Figure 6A, there was no significant difference in the height of the growth plates between the N and M group, indicating that the high-fat diet did not induce significant alteration in serum calcium levels. The EH group exhibited a significant increase in serum calcium levels compared to the M group (*p* < 0.01), indicating that high-dose EYG NPs promoted the absorption of calcium. However, no significant differences in serum phosphorus levels were observed among the groups (*p* > 0.05; Figure 6B).

Total ALP is commonly recognized as a biochemical indicator of bone formation [35]. In early childhood and adolescence, serum ALP levels are positively correlated with growth rate, making it a reliable marker for monitoring skeletal development [36]. As illustrated in Figure 6C, a significant reduction in ALP levels was observed in the M group relative to the N group (*p* < 0.01). While the EH group significantly enhanced ALP activity compared to the M group (*p* < 0.05).

In this study, a high-fat diet induced obesity model was established in mice starting at 6 weeks of age to evaluate the effects of EYG NPs on longitudinal bone growth. At the beginning of the experiment, the age of mice was approximately equivalent to 12–14 years of age in humans, representing the adolescent stage; after 12 weeks of intervention, the age of mice corresponded to the human age of 20–25 years, representing early adulthood [37,38]. Currently, most studies on longitudinal bone growth are based on adolescent rodent models with experimental durations of only 3–4 weeks [34,39]. While our study spanned both adolescence and early adulthood (12 weeks), enabling dynamic observation of the developmental transition from the growth phase to skeletal maturity.

Compared with the N group, the M group showed increase in body length and decrease in serium ALP, but no significant differences in BMD, BMC, serum calcium, or phosphorus levels, indicating that there was no significant difference in bone metabolism indicators between obesity and normal group during experiment period. After intervention with EYG NPs, tibial length, growth plate height, ALP activity, and serum calcium levels of the EH group were significantly increased compared with the M group. Previous studies have shown that each egg yolk contains 20% EYGs [40]; thus, one egg of 60 g contains approximately 4 g of EYGs. According to Table S4, a mouse of 40 g (body weight) consumed an average 2.70 g of diet per day, this corresponds to an intake of 641 mg/kg.bw of EYG NPs in EL group (0.95% EYG NPs in diets), which is equivalent to a daily intake of about 1.5 eggs for a 60 kg human. The results suggested that a daily intake of EYG NPs, at a dose equivalent to 2 to 3 eggs, had the potential to promote longitudinal bone growth, as well as to maintain normal bone metabolism during the adolescent stage.

Adequate dietary intake of calcium (Ca) and phosphorus (P) plays an important role in promoting bone mineralization and maintaining skeletal strength. Lee et al. [41] demonstrated that higher dietary Ca and favorable Ca:P ratios were associated with greater femoral-neck bone mineral density through population-level observational analysis. Wei et al. [42] conducted a cross-sectional study and found that higher dietary intakes of Ca and P were positively associated with increased bone mineral density in adults. Collectively, these findings indicated that maintaining an optimal Ca/P ratio (approximately 1:1–2:1) in the diet is critical for achieving optimal bone development and preserving skeletal health. In this study, although the EYG NPs have a relatively low Ca content (0.660 mg/g) and a high P content (17.30 mg/g), given their low proportion in the mouse diet formula (0.95% for EL, 1.9% for EH), the Ca and P contents in the diets of the EL and EH groups did not vary significantly (Table 4). The Ca/P ratio shifted from 2.0 in the M group to 1.9 in the EL group and 1.8 in the EH group, and all these ratios fell within the range of 1:1 to 2:1. Therefore, it was speculated that the likelihood of additional P load leading to changes in blood Ca and P levels (Figure 6) is relatively low. Liu et al. [43] reported that PPPs could improve BMD and promote calcium accumulation in mice bones. Zhao et al. [44] prepared a PPP–Ca complex and found it effectively enhanced calcium supplementation and improved femoral BMD in mice. Choi et al. [45] further demonstrated that a PPP-rich diet significantly increased BMD and calcium–bone binding in SD rats. Due to the high content of PV in EYG and the relatively high release of PPPs caused by EYG NPs, EYG NPs were found to effectively promote longitudinal bone growth during the adolescent stage in obesity mice. However, no comparison was made regarding the effects before and after the modification of EYGs, then the effect of nanoformulation was not clear because the animal experiments did not incorporate a non-nanomaterial group.

## 4. Conclusions

Due to the disadvantage of low solubility of egg yolk granules, in this study, the egg yolk granules were modified by combining (NaPO_3_)_6_ with ultrasound. This process caused the egg yolk granules to disintegrate and form nanoparticles through self-assembly, thereby increasing their solubility and subsequently enhancing the digestibility of internal phosvitin. Based on long-term dynamic experimental observations, the body length of obesity model increased, while serum ALP activity decreased, and no significant differences in bone mineral density were observed compared with the Normal group. Furthermore, Long-term intervention with egg yolk granule nanoparticles at high dose during the adolescent stage and early adulthood promoted longitudinal bone growth of obese mice, as evidenced by increased tibial length, elevated serum ALP activity, and enhanced growth plate height, while maintaining bone mineral density and normal bone metabolism. These results suggested the promising potential of egg yolk granule nanoparticles on promoting the bone growth of adolescents with obesity. However, as this study also has some limitations, such as single-sex design, absence of mechanistic endpoints, and lack of raw egg yolk granule control, further mechanistic studies and clinical trials are needed in the future.

## Figures and Tables

**Table 1:** Nutrient content of EYGs.

Nutrients	Mass Fraction (%)				
	Moisture	Protein	Fat	Ash	Cholesterol
	0.23 ± 0.02	53.62 ± 1.25	37.24 ± 0.61	4.88 ± 0.11	0.92 ± 0.02

**Table 2:** Amino acid composition of EYGs.

Amino Acid Species	Relative Content (g/100 g)	Amino Acid Species	Relative Content (g/100 g)
Asp	4.50	Tyr	3.63
Glu	3.00	Val	6.67
Ser	7.55	Met	3.70
Gly	6.19	Cys	2.10
His	6.76	Ile	1.01
Thr	5.15	Leu	16.82
Arg	4.72	Phe	3.83
Ala	9.40	Lys	9.50
Pro	5.47		

**Table 3:** Soluble protein content, Ca^2+^ concentration, Phosphorus content and Digestibility of egg yolk granules treated with (NaPO_3_)_6_ combined with ultrasound.

Group	Soluble Protein (mg/mL)	Ca^2+^ (μg/mL)	Phosphorus Content Before Digestion (mg/g)	Phosphorus Content After Digestion (mg/g)	Digestibility (%)
C	0.110 ± 0.004 ^e^	0.27 ± 0.03 ^d^	14.10 ± 1.21 ^b^	12.4 ± 0.31 ^b^	12.04 ± 2.22 ^b^
C′	0.593 ± 0.002 ^d^	2.43 ± 0.01 ^c^	/	/	/
P	0.692 ± 0.005 ^c^	3.28 ± 0.03 ^a^	/	/	/
U	0.733 ± 0.011 ^b^	2.59 ± 0.03 ^b^	/	/	/
PU	0.784 ± 0.016 ^a^	3.29 ± 0.03 ^a^	17.30 ± 0.28 ^a^	14.38 ± 0.49 ^a^	16.88 ± 2.85 ^a^

Values are expressed as mean ± SD (*n* = 3). Statistical analysis was performed using one-way ANOVA followed by Tukey’s post hoc test. Different letters within the same column indicate significant differences (*p* < 0.05).

**Table 4:** The calcium and phosphorus content in the diets/EYG NPs.

Group	EYG NPs Content (%)	Calcium Content (mg/g)	Phosphorus Content (mg/g)	Ca/P Ratio
N	0	5.700	2.80	2.0:1
M	0	7.800	3.80	2.0:1
EL	0.95	7.706	3.96	1.9:1
EH	1.9	7.612	4.13	1.8:1
EYG NPs	/	0.660	17.30	0.04:1

## Data Availability

The original contributions presented in the study are included in the article/Supplementary Materials. Further inquiries can be directed to the corresponding author.

## Supplementary Materials

The following supporting information can be downloaded at: https://www.mdpi.com/article/10.3390/foods14173109/s1, Table S1: Design and grouping of animal experiments; Table S2: Composition of experimental diets; Table S3: Energy intake of experimental diets; Table S4: The food intake of the experimental mice.
