# Supplementary material for: Egg Yolk Granule Nanoparticles Promote Longitudinal Bone Growth in HFD-Obese Mice
Source: Foods. 2025 Sep 5;14(17):3109. doi: 10.3390/foods14173109 (PMC12427661; doi:10.3390/foods14173109)
Supplement: Supplementary file 1 [file foods-14-03109-s001.zip › foods-3819078-supplementary.pdf]

Supplementary materials

Table S1. Design and grouping of animal experiments.

Group	Number	Diet
N: The normal group	10	Low-fat diet (10% total fat content)
M: The model group	10	High-fat diet (60% total fat content)
EL: Low-dose EYG NPs group	10	High-fat diet supplemented with 0.95% EYG NPs
EL: High-dose EYG NPs group	10	High-fat diet supplemented with 1.90% of EYG NPs

Table S2. Composition of experimental diets.

Ingredients (g/kg)	N	M	EL	EH
EYG NPs	0	0	9.5	19
EYG/Cur NPs	0	0	0	0
Casein	189.56	258.46	258.46	258.46
L-cystine	2.84	3.88	3.88	3.88
Corn starch	479.79	0	0	0
Maltodextrin	118.48	161.53	161.53	161.53
Sucrose	73.74	88.91	88.91	88.91
Cellulose	47.39	64.61	64.61	64.61
Soybean oil	23.70	32.31	32.31	32.31
Lard	18.96	316.60	316.60	316.60
Mineral mix	9.48	12.92	12.92	12.92
$CaHPO_4$	12.32	16.80	16.80	16.80
$CaCO_3$	5.21	7.11	7.11	7.11
$C_6H_5K_3O_7$	15.64	21.32	21.32	21.32
Vitamins	0.95	12.92	12.92	12.92
$C_9H_{19}NO_7$	1.90	2.58	2.58	2.58
Blue food dye	0.01	0.06	0.06	0.06
Yellow food dye	0.04	0	0	0
Total	1000	1000	1009.5	1019

Table S3. Energy intake of experimental diets.

N			M		
Product #D12450J	gm%	kcal %	Product #PD6001	gm%	kcal%
Protein	19	20	Protein	26.2	20
Carbohydrate	67.5	70	Carbohydrate	26.3	20
Fat	4.3	10	Fat	34.9	60
Total		100	Total		100
kcal/gm	3.84		kcal/gm	5.24	
Ingredient	gm	kcal	Ingredient	gm	kcal
Casein, 80 Mesh	200	800	Casein, 80 Mesh	200	800
L-Cystine	3	12	L-Cystine	3	12
Corn Starch	506.2	2024.8	Maltodextrin 10	125	500
Maltodextrin 10	125	500	Sucrose	68.8	275.2
Sucrose	77.8	311.2	Cellulose, BW200	50	0
Cellulose, BW200	50	0	Soybean Oil	25	225
Soybean Oil	25	225	Lard	245	2205
Lard	20	180	Mineral Mix, S10026	10	0
Mineral Mix, S10026	10	0	DiCalcium Phosphate	13	0
DiCalcium Phosphate	13	0	Calcium Carbonate	5.5	0
Calcium Carbonate	5.5	0	Potassium Citrate, 1 H2O	16.5	0
Potassium Citrate, 1 H2O	16.5	0	Vitamin Mix, V10001	10	40
Vitamin Mix, V10001C	1	4	Choline Bitartrate	2	0
Choline Bitartrate	2	0	FD&C Blue Dye #1	0.05	0
FD&C Blue Dye	0.01	0			
FD&C Yellow Dye	0.04	0			
Total	1055.05	4057	Total	773.85	4057.2

EL			EH		
Product #PD6001-A0.95	gm%	kcal%	Product #PD6001-A1.9	gm%	kcal%
Protein	26.2	20	Protein	26.2	20
Carbohydrate	26.3	20	Carbohydrate	26.3	20
Fat	34.9	60	Fat	34.9	60
Total		100	Total		100
kcal/gm	5.24		kcal/gm	5.24	
Ingredient	gm	kcal	Ingredient	gm	kcal
Casein, 80 Mesh	200	800	Casein, 80 Mesh	200	800
L-Cystine	3	12	L-Cystine	3	12
EYG NPs(0.95%)	**7.45**	**0**	EYG NPs(1.90%)	**15**	**0**
Maltodextrin 10	125	500	Maltodextrin 10	125	500
Sucrose	68.8	275.2	Sucrose	68.8	275.2
Cellulose, BW200	50	0	Cellulose, BW200	50	0
Soybean Oil	25	225	Soybean Oil	25	225
Lard	245	2205	Lard	245	2205
Mineral Mix, S10026	10	0	Mineral Mix, S10026	10	0
DiCalcium Phosphate	13	0	DiCalcium Phosphate	13	0
Calcium Carbonate	5.5	0	Calcium Carbonate	5.5	0
Potassium Citrate, 1 H2O	16.5	0	Potassium Citrate, 1 H2O	16.5	0
Vitamin Mix, V10001	10	40	Vitamin Mix, V10001	10	40
Choline Bitartrate	2	0	Choline Bitartrate	2	0
FD&C Blue Dye #1	0.05	0	FD&C Blue Dye #1	0.05	0
Total	781.3	4057.2	Total	788.85	4057.2

Table S4. The food intake of the experimental mice.

The average daily food intake (g) of each mouse	N	M	EL	EH
1st week	3.23	2.82	3.01	2.86
2nd week	3.03	2.47	2.53	2.54
3rd week	3.06	2.58	2.71	2.79
4th week	2.95	2.54	2.69	2.74
5th week	2.96	2.72	2.71	2.72
6th week	3.01	2.74	2.86	2.77
7th week	3.01	2.62	2.76	2.86
8th week	2.97	2.51	2.78	2.86
9th week	3.05	2.71	2.62	2.71
10th week	2.88	2.76	2.70	2.68
11th week	2.86	2.73	2.44	2.55
12th week	2.96	2.78	2.58	2.52
Average	3.00	2.67	2.70	2.72
